# Using participatory approaches with children to better understand their physical activity behaviour

**DOI:** 10.1177/0017896918759567

**Published:** 2018-04-02

**Authors:** Felicity ZL Hayball, Charlotte Skau Pawlowski

**Affiliations:** a Department of Nutritional Sciences, School of Biosciences and Medicine, Faculty of Health and Medical Sciences, University of Surrey, Guildford, UK; b MRC/CSO Social and Public Health Sciences Unit, University of Glasgow, Glasgow, UK; c Department of Sports Science and Clinical Biomechanics, University of Southern Denmark, Odense, Denmark

**Keywords:** Children, outdoor environments, participatory approaches, physical activity behaviour, visual data

## Abstract

**Aims and objectives:**

The importance of childhood physical activity is widely recognised. Helping children to articulate their opinions is a crucial factor in improving their health and well-being, yet the field is predominantly focused on adult-led quantitative methods and lacks deeper understanding from a child perspective.

**Methods:**

This paper draws on experiences from a Danish study in which children depicted their physical activity behaviour in go-along group interviews in schoolyards (*n* = 111), and a Scottish study in which children photographed or drew meaningful places and discussed physical activity in these places (*n* = 25).

**Results:**

The benefits and challenges associated with using participatory methods to understand how children perceive the environment in relation to their physical activity behaviour are described.

**Conclusion:**

Findings contribute to the literature by suggesting that participatory approaches are valuable in capturing children’s perceptions of physical activity behaviour in outdoor environments.

## Introduction

The importance of physical activity (PA) for children’s physical, social and mental health is widely recognised (Andersen et al., 2004; Janssen and Leblanc, 2010). Nevertheless, a high number of school-aged children in countries such as Denmark and Scotland do not reach the recommended level of 60 minutes of moderate-to-vigorous physical activity (MVPA) per day. Among Danish children, 21%–40% adhere to the global guidelines, whereas in Scotland, a maximum of 20% of children meet these PA guidelines (Trembley et al., 2016).

Independent outdoor PA has come to the fore as a way to increase the children’s PA and improves their health (Brockman et al., 2010; Ergler et al., 2013). Studies have revealed that time spent outdoors engaged in independent active play correlates positively with higher objectively measured PA levels (Cleland et al., 2008; Mackett and Paskins, 2008). However, children’s outdoor play has declined both in school and after school hours (McBride, 2012; Massey et al., 2017). Environmental variables are increasingly investigated as determinants of children’s outdoor play practices (Ergler et al., 2013; Sterdt et al., 2014), but little is known about how children perceive the environment in which independent outdoor PA occurs.

Allowing children to voice their perceptions is important in order to understand and improve the health of children (Darbyshire et al., 2005). A research perspective supportive of this approach is ‘the new social studies of childhood’ (Holloway and Valentine, 2000), developed as a result of the general neglect of childhood in earlier sociological research. Hitherto, children had largely been seen as adults in the making rather than children in a state of being; as incomplete and incompetent, and if studied at all, through the lens of adults. From the late 1990s onwards, this new approach sought to recognise children as active agents, competent social actors and individuals with rights (Woodhead and Faulkner, 2008). However, in medical and health science, it is still the case that research methods are predominantly adult-led and quantitative (Raphael, 2000). This may be valuable in identifying PA prevalence and identifying associations, but these methods do not provide contextual understanding and cannot readily explain why some children are more physically active than others (Noonan et al., 2016; Pawlowski et al., 2016a).

Researchers exploring childhood PA are increasingly aware that there are gaps in understanding of children’s perspectives and behaviours (Ross and Francis, 2016) and using qualitative, participatory approaches in line with ‘the new social studies of childhood’ would help to fill this gap. Participatory methods help to ensure children are at the centre of the data collection and analysis and can help to enhance credibility and trustworthiness in the data due to the minimisation of researcher bias. They can also help children to voice their perceptions in alternative ways due to the fact that verbal communication may not be the preferred mode of communication for all children (Noonan et al., 2016).

A number of researchers in the field of childhood PA have used participatory approaches to understand children’s PA behaviour (Cammisa et al., 2011; Darbyshire et al., 2005; Knowles et al., 2013; Lassetter et al., 2015; MacDougall et al., 2004; Noonan et al., 2016; Ross and Francis, 2016; Saimon et al., 2015; Veitch et al., 2008). However, an in-depth discussion of participatory approaches is needed to better understand their strengths and limitations (Darbyshire et al., 2005; MacDougall et al., 2004; Noonan et al., 2016).

Using two of our studies as examples, this paper will examine the benefits and challenges associated with using a selection of participatory methods (go-along, photographs and drawings) to understand 10- to 11-year-old children’s perceptions of their PA behaviour in environments suited for independent outdoor PA. The studies focused in this paper, conducted by the authors, were carried out in Denmark and Scotland, respectively (Table 1)

**Table 1.** Summary of the two studies.

	Study 1	Study 2
Location	Denmark	Scotland
Setting	The outdoor environment at 17 nationally spread schools	The outdoor environment in neighbourhoods within the Glasgow and Edinburgh area
Foci	Children’s perceived factors in the schoolyard environment influencing their PA behaviour during recess	Physical and social affordances’ influence on children’s place preference
Period	April–June 2013	May–July 2015
Participants	*N* = 111	*N* = 25
	53 boys, 58 girls	12 boys, 13 girls
	10- to 11-year olds	10- to 11-year olds
Methods	Go along semi-structured interviews Brainstorming sessions including post-it note activity	Photovoice Drawings Focus groups Interviews
Analysis	Deductive thematic analysis for interview data Content analysis for post-it notes	Participant analysis for visual data Concurrent inductive and deductive thematic analysis for verbal data

PA: physical activity.

## Study 1 – using go-along group interviews

The first study took place in the outdoor environment at 17 Danish schools included in a schoolyard intervention study. The schools varied in geographic location, school type, number of pupils, socioeconomic status (based on parental income), square metres of schoolyard per child, recess rules and number of play facilities (Pawlowski et al., 2014). The research question being asked was how children perceive factors in the schoolyard environment influencing their PA behaviour during recess.

To understand the children’s interaction with the physical recess environment, go-along group interviews were utilised in this study. The go-along method involves a combination of in-depth interviews and observations conducted by researchers while participating in a tour guided by informants around their ‘natural’ location (Kusenbach, 2003). By asking questions and observing, the interviewer is able to explore informants’ perceptions, experiences, interpretations and practices within this environment (Carpiano, 2009). In the Activating Schoolyards Study, children took the interviewer on a walking tour around their schoolyard.

Data were collected during a one-day visit to each of the 17 schools between April and June 2013. Approximately, three boys and three girls aged 10–11 years old in each school were purposely sampled with help from the school principal or a designated teacher who knew the children and could help recruit children with diverse characteristics to ensure variation in gender, social backgrounds and PA levels, to allow for contrasting opinions (Krueger and Casey, 2002; Morgan, 1997). A total of 17 go-along group interviews (one at each school) were conducted. In total, 111 children (53 boys and 58 girls) participated in a go-along group interview. The group-size ranged from 5 to 10 participants. The go-along group interviews lasted for approximately 60 minutes and were conducted during school hours. They were semi-structured in design, using both prepared and non-consistent ad hoc questions. The prepared questions helped keeping focus at the explored environment. The more ad hoc questions were posed to allow for further exploration of the children’s answers or acts in the environment. Questions during the walk included, for example, ‘What do you think about your schoolyard?’, ‘Where are you the most during your recess?’ and ‘What are your experiences around recess?’ Interviews were videoed using an iPad mini to record interactions and to document who said what (Kvale and Brinkmann, 2009). At the end of the go-along interview, an open brainstorm session was conducted where the groups were told to write down all environmental factors influencing their recess PA that they could think of on post-it notes.

In the first phase of the analysis, all the post-it notes with the environmental factors were ranked based on the number of times they were mentioned using a content analysis (Elo and Kyngäs, 2008). This ranking was created to guide the development of a set of prioritised factors as perceived by the children. Next, a deductive thematic analysis of the transcripts from the go-along group interviews (recorded on the iPad mini) was conducted to produce an in-depth description and understanding of ranked factors as perceived by the children (Braun et al., 2016). Phrases from transcripts that referred to the children’s perceived factors influencing their recess PA were highlighted and grouped, from which themes and subthemes emerged. From this analysis, several environmental factors in the schoolyard environment were identified as influencing children’s recess PA, such as space and place experiences, play facilities, conflicts, the use of electronic devices and the weather. Further discussion of results from study 1 can be found elsewhere (Pawlowski et al., 2014, 2016b).

## Study 2 – using photographs and drawings

Study 2 took place across the central belt of Scotland and included the wider conurbations of Glasgow and Edinburgh. Children were recruited from an ongoing Scottish longitudinal birth cohort study – Growing Up in Scotland (GUS). Invitation and information letters were sent to GUS participants to enquire whether they would be interested in taking part in a qualitative study exploring PA behaviours in their local environment.

Twenty-five 10- to 11-year-old children (12 boys and 13 girls) from varying levels of area deprivation and rurality took part in the study. In determining urban and rural grade, the authors used the Scottish Government sixfold Urban–Rural Classification. To determine area deprivation, the authors used the Scottish Index of Multiple Deprivation (SIMD). The research questions asked were the following: how might physical and social affordances influence children’s place preference, and does this differ depending on area deprivation and degree of urbanicity? Data were collected between May and July 2015.

A key element of this study was to understand the environment from the child’s point of view. Visual material can help understand a complex phenomenon, such as PA behaviour (Cooper et al., 2017). Visual data, in particular, have come to play a key role in various ecological studies of child–environment relationships (Jorgenson and Sullivan, 2010; Rasmussen, 2004). Therefore, photos and drawings produced by the children were selected as a means to develop a deeper understanding of the children’s PA behaviour. Children were each given a disposable camera and a sketchbook and asked to document locations and/or features in their neighbourhood that they felt influenced their time spent outside. Once the visual data collection was complete, the children could choose to participate in either a single interview (*n* = 11) lasting for approximately 45 minutes or a focus group (*n* = 10 in three focus groups) lasting for approximately 60 minutes. A mixture of single interview and focus groups were used in an attempt to make children feel comfortable. Four participants dropped out between the visual and verbal data collection periods.

The interviews and focus groups commenced with the participant analysis of the visual data. Each child was given a six box grid, four of the boxes were labelled as follows: ‘places I like to go’, ‘places I don’t like to go’, ‘things I like to see’ and ‘things I don’t like to see’. Children were asked to place their photographs/drawings into the box they felt represented their visual data. If some or all of their visual data did not fit into the labelled boxes, there were two non-labelled boxes that they could label themselves. Once the participant analysis had been completed, the interview/focus group began. Both the interviews and focus groups were semi-structured – discussion in both interviews and focus groups was prompted by the visual data. Interviews and focus groups were recorded and transcribed verbatim and accuracy was checked by two researchers.

The analytic framework for the study involved concurrent inductive and deductive thematic analysis (Fereday and Muir-Cochrane, 2006). The transcripts of the focus groups were read by the lead author who then sorted the verbal data into raw codes. These raw codes were identified for patterns relating to the research aim; raw codes that followed similar patterns were placed together and arranged into first-order themes. The same process then took place with the first-order themes to place them into second-order themes, which were then grouped together to form overall themes. The overall themes were further condensed into two groups – physical affordances and social affordances.

**Figure 1.**
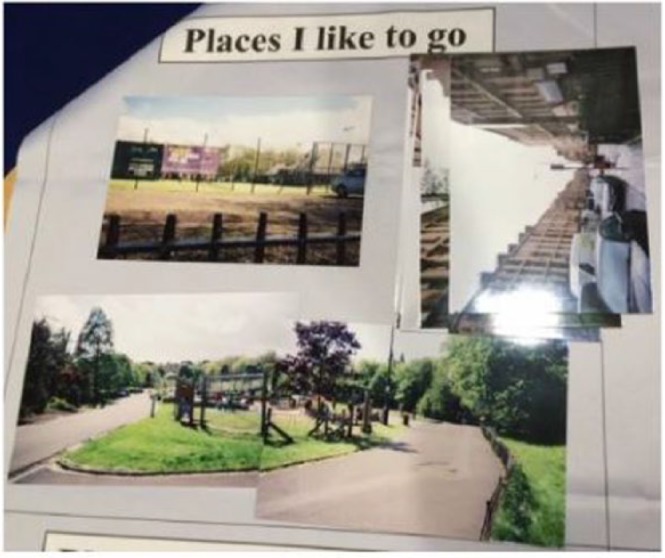
Children’s photographs placed in the ‘Places I like to go’ box during the participant analysis.

**Figure 2.**
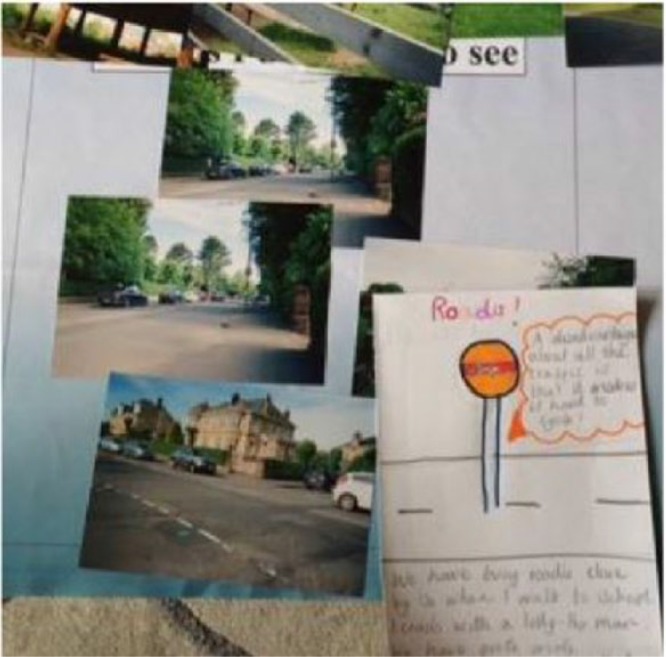
Children’s photographs placed in the ‘Things I like to see’ box during the participant analysis.

**Figure 3.**
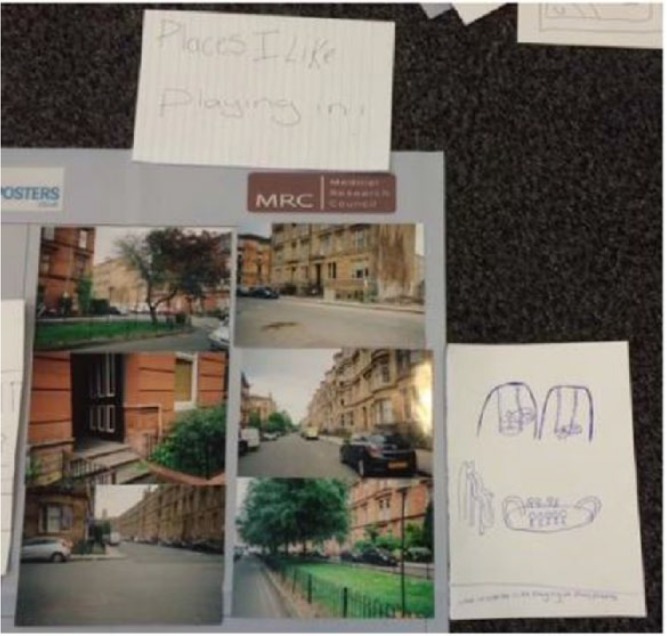
Children’s photographs placed in the self-labelled ‘Places I like playing in’ box during the participant analysis.

From the analysis, it emerged that children from different levels of rurality experienced the environment differently. One of the key findings from this study was that the urban-based children were more likely to be influenced by the social environments and primarily friendship groups, while rural-based children appeared to be influenced more by the physical environment. The visual and the verbal data supported these conclusions suggesting continuity between the methods. Examples of these can be seen in Figures 1–3.

## Ethical considerations

Information about the studies was provided to all involved persons prior to data collection, that is, children, parents/guardians and also to school principals and class teachers in the first study. In study 1, teachers handed out informed consent forms for the parents/guardians of the invited children to complete. In study 2, consent forms were given to the parents/guardians and the children. All children were informed that they could withdraw at any time from the respective study. In the second study, the children were told to try and avoid taking pictures of other children but that any faces would be blacked out during the reproduction of photographs. The children were also given a telephone number to call if anyone had any questions over why they were taking photographs.

According to the Danish National Committee on Health Research Ethics, formal approval was not required for the first study, as the study was not a biomedical research project. Data management and security with regard to this study was approved by the Danish Data Protection Agency (2013-41-1900). Study 2 was approved by the Research Ethics Committee (application number: 400130195).

## Discussion

What then is the value of using these methods when aiming to understand children’s PA behaviour, and what can be learned from employing a participatory approach with children?

### Reducing power imbalances

One of the main challenges when working with children is to diminish the power imbalance between the adult researcher and the child. An unequal power balance can cause discomfort for the child and may inhibit expression (Woodhead and Faulkner, 2008). By providing children with a wider range of methodological options, and options that place them in control of the data collection, the child may feel more comfortable and express themselves with more honesty and openness (Noonan et al., 2016).

In the first study, the children took the lead and served as tour guides conducting go-along interviews. This ‘show and tell’ method provides children with an alternative way of communicating their perceptions and as such is a more inclusive research approach (Carpiano, 2009). As it is the children who guide the tour, they help control the interview. Moreover, the proximity of schoolmates when conducting the go-along interviews in groups made the adult interviewer less visible and reduced the discomfort that some of the children may have felt about being followed as asked about their PA behaviour (Kusenbach, 2003).

Using photographs and drawings, as in study 2, also reduces the authority of the adult researcher and can empower participants (Rasmussen, 1999). We found that designing the interview/focus group guide around the grid with photographs and drawings provided the children with greater control over the conversation and enabled a more focused discussion.

### Recall of memories

Another strength of the participatory methods used was that they could help children express memories of their PA behaviour. When using the go-along interview method, for example, the children often recalled memories of how they had played at specific places when moving around in their recess environment. Similarly, in study 2, it was found that using photographs in the interview triggered memories enabling children to provide nuanced dimensions of their neighbourhood experiences. Using participatory approaches to aid memory recall has been documented by other researchers in the research field of children (Clark-Ibanez, 2004; Darbyshire et al., 2005; Kusenbach, 2003; Miller, 2014).

### An opportunity to talk

Although quantitative methods are frequently used with children, these methods do not allow children to express and discuss their perceptions of PA in the same way as interviews and focus groups (Darbyshire et al., 2005; Docherty and Sandelowski, 1999).

In both of the studies documented here, it was found that children appreciated the opportunity to discuss their PA perceptions in their neighbourhood and schoolyard and felt valued that someone had taken the time to listen to what they had to say (Agar, 1996). In addition, offering the children a choice encouraged more relaxed and forthcoming participation. In study 2, the children were given the choice of taking part in a focus group or a single interview. Some children noted that they would prefer one over the other, some did not have a preference and some stated they would only be happy taking part in one. By giving children control, the researchers hoped data collection would be a more comfortable experience for the child.

Verbal communication may not be the preferred mode of communication for all children (Noonan et al., 2016). In study 1, requesting the children to show the researcher around their schoolyard seemed to increase motivation to talk, especially among the more silent children. Using interactive and creative methods, such as taking photos and drawing in study 2, also worked as techniques to engage the more reserved children.

### Research freedom

Mauthner (1997) has suggested that researchers should give children the freedom to set their own agenda. Study 1 treated the children as experts on their own lives by enabling children to be tour guides during the go-along interview. It was up to the children what they wanted to show the researcher and talk about in the outdoor school environment. Study 2 demonstrated a similar commitment by designing the interview guide based on each child’s visual data. Furthermore, it was up to the children what they chose to take photographs of and what they chose to draw. This meant that each interview was guided by what the child felt was important, thereby making each interview guide unique to that child.

### Reducing researcher bias

Children may view the world differently to adult researchers. Therefore, misinterpretation by researchers can occur when using traditional interviews and focus groups. It has also been argued that visual data collected by children are open to misinterpretation (Gabhainn and Kelleher, 2002). However, participatory research that builds on visual data combined with verbatim accounts has been found to reduce this (Hurworth, 2003).

In study 2, no attempt was made to interpret the photographs and drawings beyond the verbatim descriptions provided by the children themselves. The researchers found that this gave control of the analysis to the participant, thereby reducing potential bias and increased the credibility of the findings. For example, in this same study, many photographs/drawings of children’s playgrounds were placed in the box ‘places I don’t like to go’. Finding that the children did not enjoy spending time at a playground may not have been discovered if an adult was analysing the visual data.

In study 1, the first step of the analysis was to group the children’s post-it notes with their identified factors influencing recess PA and then rank them based on the number of times specific factors were mentioned by the children. This ensured the analysis was guided by the children’s own prioritisation of perceived factors, reducing researcher bias. Thus, the analysis in both studies was not solely dependent upon the researcher’s interpretation of the data, reducing the risk of misinterpretation, improving data credibility and enhancing confidence in the findings (Noonan et al., 2016).

### Contribution to literature and policy implications

Past studies in childhood PA have frequently used quantitative methods, which is valuable in providing prevalence levels of PA. However, what such research does not provide is deep contextual understanding (Noonan et al., 2016; Pawlowski et al., 2016a). For example, Johnson et al. (2010) used pedometers to compare step counts in children and found that children from suburban and rural areas accumulated significantly more steps per day than children living in more urban areas. Moore et al. (2013) using accelerometer data found that young people living in urban areas had higher mean levels of MVPA compared to rural youth. These studies collected different types of PA information; yet, neither provides an explanation as to why the children from different settings accrued more or less PA. In contrast, more participatory approaches can help researchers understand why the children chose to visit or avoid particular locations in their outdoor environment, how and why the children chose to interact with school-based playgrounds the way they do and which features in the environment encourage PA and the reasoning behind this.

Here, findings suggested that children perceive the environment differently, which may affect how they use the environment for PA. In study 2, the rural children spoke more of walking further to play spaces, while the photos from the children living in urban areas were of places nearby. In addition, the children living in urban areas spoke more of friends being influential with respect to their PA behaviour. Although more research is needed to explore urban/rural comparisons, this shows that participatory research is necessary to gain a better understanding of children’s PA behaviours.

Furthermore, data collected by participatory methods can more readily be shared with key decision-makers. Findings from study 1 linked closely to a schoolyard intervention by providing the school principal, designers and architects with knowledge about factors influencing children’s PA behaviour during recess. Findings were easily used as the children clearly articulated how perceived barriers created significant obstacles to establishing healthy behaviours during recess. Moreover, children themselves suggested ways of mitigating some of these barriers (e.g. by suggesting restrictions for use of mobile phones during recess). Similarly, children in study 2 provided their opinions on how to create a more playful environment, such as by adding rain covers to playground equipment and signs written by children to stop adults smoking in play areas. Study 2 was also associated with a large-scale longitudinal study in Scotland exploring children’s health. Findings from the study were used in public engagement events to inform the public about how the environment can be influential to children’s health.

In participatory research studies such as these, perceptions come from the principal source – the child who is treated as the expert and central to the research process. Secondary sources such as teachers and parents can accidently misinterpret children’s perceptions or give a false view of the child’s perceptions. For example, Eyre et al. (2014) found that parents felt the main barrier to childhood PA was neighbourhood safety; however, in study 2, very few children, and none from the areas of high deprivation, reported that safety was a concern or that it prevented them from being active. Safety concerns may influence the independent mobility that children are given by their parents; however, it may not be a barrier that the children themselves perceive. Future research that aims to understand the lives of children should therefore endeavour to involve children as much as possible in the research process.

### Lessons learned

The participatory research approaches used within the two studies described here are still relatively new within the field of childhood PA. What then can be learned from their application?

While both studies helped to reduce the authority of ourselves as adult researchers and helped children to express their opinions, it is important to be aware of the power imbalance that remains between the participants and researchers. Due to differences between adults and children in terms of cognitive and communicative maturity, power, and physical size, equality is difficult to reach (Eder and Corsaro, 1999; Mandell, 1988). While the children were not seen solely as a data source, they were also not considered full researchers. Instead, they were perhaps more like what Hart (1992) defines as consultants. They understood the process and their opinions were treated seriously.

Several researchers have chosen to use parental proxies in studies concerning childhood PA (Trigwell et al., 2015). If the aim of the study is to understand how parents perceive the child’s PA behaviour or to understand the potential restrictions they place on their child’s PA, then this method does have relevance. However, some researchers do not involve children at all in research, even when the topic directly relates to their health. For example, while exploring child perceptions of how the built environment can influence their PA, Teedon et al. (2014) chose not to engage with children because the ‘children might find the connections between environment and health too abstract and thus difficult to deal with’ (p.51). In contrast, we feel that given the depth and relevance of the data elicited in our studies, there is clear evidence to support children’s direct involvement in research, when it is facilitated in a suitable manner.

A perhaps unexpected finding from our research was the difficulties some adults had in letting us interview children on their own. An existing power relation between children and their gatekeeper can be a barrier to free speech and may influence what children choose to say (Carstensen et al., 2013). In study 1, it was difficult to convince one teacher that she should not take part in the interviews with the children. In study 2, some parents were surprised at being asked to leave during data collection. Such responses can create a degree of awkwardness between the researcher and the gatekeeper. It is important, therefore, that the researcher asks the child if they are comfortable being left alone. If the child is happy to be one-on-one with the interviewer, the interviewer should feel comfortable asking the gatekeeper to leave the interview space; if they do not wish to leave, the researcher would need to discuss with them their concerns or apprehensions and try to reach the best solution. One potential way to overcome this difficulty might be to discuss with the gatekeepers prior to the interview, why they will likely be asked to leave. This could be achieved by adding this information to the briefing provided to the gatekeeper.

## Conclusion

This article is based on a synthesis of results from two studies using different participatory approaches. The methodological factors that informed the design of our studies are described and discussed, providing the transparency to enable future studies to adopt similar approaches. On the basis of our experience, we strongly believe that child participatory approaches are valuable in order to capture children’s perceptions of their behaviour in outdoor environments. The benefits are numerous and include reducing the power imbalance between the adult researcher and the child, memory recall; reducing researcher bias; and avoiding the misrepresentation of data. In addition, the child is provided with the opportunity to discuss matters in alternative ways and has the freedom to set their own research agenda. Giving children the freedom to share their perceptions using participatory techniques allows for a deeper understanding of matters influencing their behaviour that might otherwise be overlooked when using survey or more traditional adult focused qualitative methods.
